# Draft genome sequences of strains *Salinicola socius* SMB35^T^, *Salinicola* sp. MH3R3–1 and *Chromohalobacter* sp. SMB17 from the Verkhnekamsk potash mining region of Russia

**DOI:** 10.1186/s40793-017-0251-5

**Published:** 2017-07-19

**Authors:** Björn E. Olsson, Ekaterina S. Korsakova, Lyudmila N. Anan’ina, Anna A. Pyankova, Olga V. Mavrodi, Elena G. Plotnikova, Dmitri V. Mavrodi

**Affiliations:** 1 University of Skövde, School of Bioscience, Skövde, Sweden; 2 Institute of Ecology and Genetics of Microorganisms, Ural Branch of the Russian Academy of Sciences, Perm, Russia; 3 Department of Biological Sciences, The University of Southern Mississippi, Hattiesburg, MS 39406 USA

**Keywords:** *Salinicola*, *Chromohalobacter*, *Halomonadaceae*, Halophile, Potash mine tailings

## Abstract

Halomonads are moderately halophilic bacteria that are studied as models of prokaryotic osmoadaptation and sources of enzymes and chemicals for biotechnological applications. Despite the progress in understanding the diversity of these organisms, our ability to explain ecological, metabolic, and biochemical traits of halomonads at the genomic sequence level remains limited. This study addresses this gap by presenting draft genomes of *Salinicola socius* SMB35^T^, *Salinicola* sp. MH3R3–1 and *Chromohalobacter* sp. SMB17, which were isolated from potash mine tailings in the Verkhnekamsk salt deposit area of Russia. The analysis of these genomes confirmed the importance of ectoines and quaternary amines to the capacity of halomonads to tolerate osmotic stress and adapt to hypersaline environments. The study also revealed that *Chromohalobacter* and *Salinicola* share 75–90% of the predicted proteome, but also harbor a set of genus-specific genes, which in *Salinicola* amounted to approximately 0.5 Mbp. These genus-specific genome segments may contribute to the phenotypic diversity of the *Halomonadaceae* and the ability of these organisms to adapt to changing environmental conditions and colonize new ecological niches.

## Introduction

The family *Halomonadaceae* encompasses a diverse group of moderately halophilic, aerobic, heterotrophic, Gram-negative bacteria that are ubiquitously present in ocean water, marine sediments, saline lakes, salt pans, salty foods, and saline soils [1]. These microorganisms can grow over a wide range of saline concentrations and received considerable attention as model systems to study the physiological and molecular mechanisms of prokaryotic osmoadaptation [2–4]. Several members of the family have been investigated because of their biotechnological potential, especially as a source of exopolysaccharides, biosurfactants, biodegradable plastics (polyhydroxyalkanoates), salt-tolerant extracellular enzymes, and compatible solutes [5]. Halomonads were used for the development of energy-efficient unsterile fermentation processes that use seawater-based growth medium [5]. Finally, certain strains of *Halomonas* can mobilize, degrade and neutralize environmental pollutants and may play a vital role in the process of remediation of contaminated saline environments [6].

Members of the *Halomonadaceae* are heterogeneous, and many currently recognized species were previously assigned to distantly related families. With the advent of molecular typing techniques in the 1990s, the group underwent a major taxonomic revision and now contains 16 validly named genera [7]. Despite the progress in understanding the diversity of these organisms, our ability to explain ecological, metabolic, and biochemical traits of halomonads at the genomic sequence level remains limited. At the time of the manuscript preparation, GenBank listed 54 genomes of bacteria of the *Halomonadaceae* family. However, two-thirds of these entries originated from strains of a single genus, *Halomonas*, while other genera within this genomically diverse group remain strongly underrepresented. Here we address this gap by presenting the description, annotation, and analysis of draft genomes of *S. socius* SMB35 ^T^, *Salinicola* sp. MH3R3–1 and *Chromohalobacter* sp. SMB17, which were isolated from soils affected by potash mine tailings in the Verkhnekamsk salt deposit area of Russia. The genome sequences of strains SMB35 ^T^ and MH3R3–1 are the first analyzed *Salinicola* genomes, and one of these genomes represents a type strain of this genus.

## Organism information

### Classification and features

**Fig. 1:**
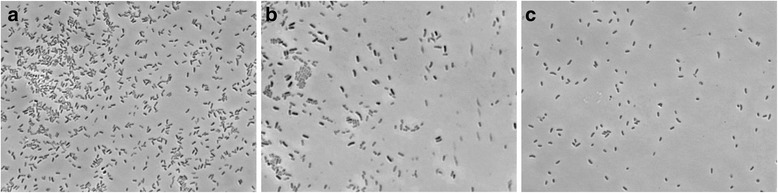
Cellular morphology of *S. socius* SMB35^T^(**a**), *Salinicola* sp. MH3R3–1 (**b**), *Chromohalobacter* sp. SMB17 (**c**) captured at 100×magnification. Bacteria were cultured in the RMM [8] supplemented with 3% NaCl, fixed and Gram-stained

*S. socius* SMB35 ^T^ (DSM 19940 ^T^), *Salinicola* sp. MH3R3–1 and *Chromohalobacter* sp. SMB17 are moderately halophilic Gram-negative, aerobic, catalase-positive and oxidase-negative, non-spore-forming bacteria that belong to the *Halomonadaceae* family in the class *Gammaproteobacteria* (Fig. 1, Tables 1, 2, and 3). Strains SMB35 ^T^ and SMB17 were isolated from saline soil collected in an area affected by potash mining activity near the town of Solikamsk, Russia. *Salinicola* sp. MH3R3–1 was isolated from the rhizosphere of salt sand-spurrey (*Spergularia salina*), a halotolerant plant commonly found around salt mine tailings in the sampled area. The isolation and subsequent maintenance were carried out at 28 °C in RMM medium [8] supplemented with 6% (*w*/*v*) NaCl, 0.5% (*w*/*v*) tryptone, and 0.25% (*w*/*v*) yeast extract. Cells of SMB35 ^T^ and SMB17 are straight rods (0.5–0.6 × 1.1–1.4 μm) with a single polar flagellum (Fig. 1). On RMM agar both strains form yellow, circular and convex colonies with smooth margins. Cells of the strain MH3R3–1 are straight motile rods that are 0.5–0.6 × 1.1–1.4 μm in size (Fig. 1). Colonies on spread plates are cream, circular and convex with an entire margin.

**Table 1:** Classification and general features of *S. socius* SMB35^T^

MIGS ID	Property	*Salinicola socius* SMB35^T^	Evidence code^a^
	Classification	Domain: *Bacteria*	TAS [41]
		Phylum: *Proteobacteria*	TAS [42]
		Class: *Gammaproteobacteria*	TAS [43]
		Order: *Oceanospirillales*	TAS [44]
		Family: *Halomonadaceae*	TAS [1]
		Genus: *Salinicola*	TAS [1, 9]
		Species: *S. socius*	TAS [9]
		(Type) strain: SMB35^T^	TAS [9]
	Gram stain	Negative	TAS [9]
	Cell shape	Rod	TAS [9]
	Motility	Motile by means of a single polar flagellum	TAS [9]
	Sporulation	Non-sporulating	TAS [9]
	Temperature range	Mesophilic	TAS [9]
	Optimum temperature	34 °C	TAS [9]
	pH range	6.0–8.0	TAS [9]
	Carbon source	D-glucose, D-galactose, D-xylose, D-maltose, D-trehalose, D-fructose, glycerol, mannitol, sorbitol, acetic acid	TAS [9]
MIGS-6	Habitat	Saline soil	TAS [9]
MIGS-6.3	Salinity	0.5–30.0%	TAS [9]
MIGS-22	Oxygen requirement	Aerobic	TAS [9]
MIGS-15	Biotic relationship	Free-living	TAS [9]
MIGS-14	Pathogenicity	Non-pathogen	
MIGS-4	Geographic location	Perm region/Russia	
MIGS-5	Sample collection	July 2002	
MIGS-4.1	Latitude	N 59.39551	
MIGS-4.2	Longitude	E 56.76835	
MIGS-4.4	Altitude	118 m	

^a^Evidence codes – IDA: Inferred from Direct Assay; TAS: Traceable Author Statement (i.e., a direct report exists in the literature); NAS: Non-traceable Author Statement (i.e., not directly observed for the living, isolated sample, but based on a generally accepted property for the species, or anecdotal evidence). These evidence codes are from the Gene Ontology project

**Table 2:** Classification and general features of *Salinicola* sp. MH3R3–1

MIGS ID	Property	*Salinicola* sp. MH3R3–1	Evidence code^a^
	Classification	Domain: *Bacteria*	TAS [41]
		Phylum: *Proteobacteria*	TAS [42]
		Class: *Gammaproteobacteria*	TAS [43]
		Order: *Oceanospirillales*	TAS [44]
		Family: *Halomonadaceae*	TAS [1]
		Genus: *Salinicola*	TAS [1, 9]
		Species: *Salinicola* sp*.*	TAS [45]
		Strain: MH3R3–1	TAS [45]
	Gram stain	Negative	NAS [45]
	Cell shape	Rod	NAS [45]
	Motility	Motile	NAS [45]
	Sporulation	Non-sporulating	NAS [45]
	Temperature range	Mesophilic	TAS [45]
	Optimum temperature	Not available	
	pH range	Not available	
	Carbon source	Not available	
MIGS-6	Habitat	Rhizosphere of *Spergularia salina*	TAS [45]
MIGS-6.3	Salinity	0–25.0%	NAS [45]
MIGS-22	Oxygen requirement	Aerobic	NAS [45]
MIGS-15	Biotic relationship	Free-living	TAS [45]
MIGS-14	Pathogenicity	Non-pathogen	
MIGS-4	Geographic location	Perm region/Russia	
MIGS-5	Sample collection	June 2011	
MIGS-4.1	Latitude	N 59.56772	
MIGS-4.2	Longitude	E 56.76833	
MIGS-4.4	Altitude	148 m	

^a^Evidence codes – IDA: Inferred from Direct Assay; TAS: Traceable Author Statement (i.e., a direct report exists in the literature); NAS: Non-traceable Author Statement (i.e., not directly observed for the living, isolated sample, but based on a generally accepted property for the species, or anecdotal evidence). These evidence codes are from the Gene Ontology project

**Table 3:** Classification and general features of *Chromohalobacter* sp. SMB17

MIGS ID	Property	*Chromohalobacter* sp. SMB17	Evidence code^a^
	Classification	Domain: *Bacteria*	TAS [41]
		Phylum: *Proteobacteria*	TAS [42]
		Class: *Gammaproteobacteria*	TAS [43]
		Order: *Oceanospirillales*	TAS [44]
		Family: *Halomonadaceae*	TAS [1]
		Genus: *Chromohalobacter*	TAS [1, 7]
		Species: *Chromohalobacter* sp.	TAS [46]
		Strain: SMB17	TAS [46]
	Gram stain	Negative	TAS [46]
	Cell shape	Rod	TAS [46]
	Motility	Motile	TAS [46]
	Sporulation	Non-sporulating	TAS [46]
	Temperature range	Mesophilic	TAS [46]
	Optimum temperature	Not available	
	pH range	Not available	
	Carbon source	Benzoic acid, p-hydroxybenzoic acid, acetic acid	TAS [47]
MIGS-6	Habitat	Saline soil	TAS [47]
MIGS-6.3	Salinity	3–30%	TAS [46]
MIGS-22	Oxygen requirement	Aerobic	NAS [46]
MIGS-15	Biotic relationship	Free-living	TAS [47]
MIGS-14	Pathogenicity	Non-pathogen	
MIGS-4	Geographic location	Perm region/Russia	
MIGS-5	Sample collection	October 2000	
MIGS-4.1	Latitude	N 59.39564	
MIGS-4.2	Longitude	E 56.76899	
MIGS-4.4	Altitude	115 m	

^a^Evidence codes – IDA: Inferred from Direct Assay; TAS: Traceable Author Statement (i.e., a direct report exists in the literature); NAS: Non-traceable Author Statement (i.e., not directly observed for the living, isolated sample, but based on a generally accepted property for the species, or anecdotal evidence). These evidence codes are from the Gene Ontology project

**Fig. 2:**
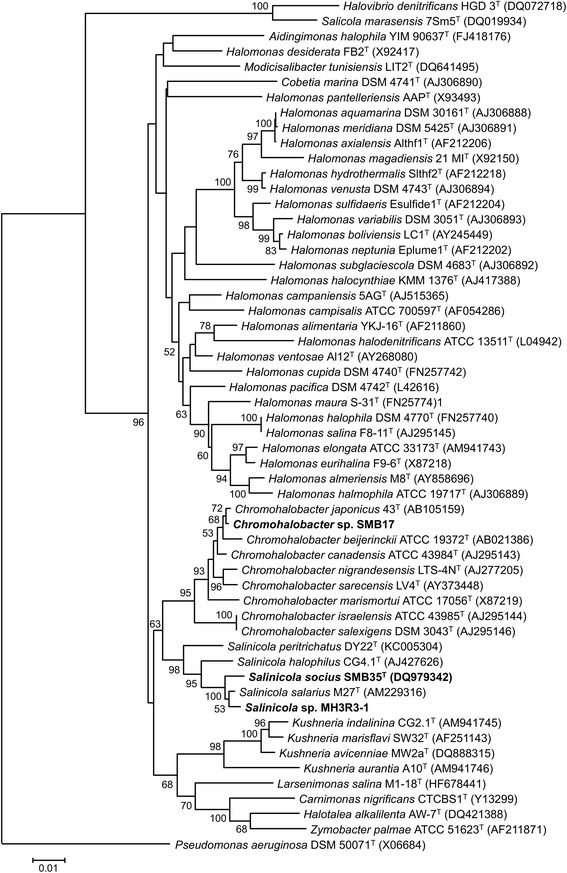
Neighbor-joining phylogeny inferred from data for 16S rRNA sequences of *S. socius* SMB35^T^, *Salinicola* sp. MH3R3–1, *Chromohalobacter* sp. SMB17 and other species of the *Halomonadaceae* family. The optimal tree with the sum of branch length = 0.95863591 is shown. The percentage of replicate trees in which the associated taxa clustered together in the bootstrap test (1000 replicates) are shown next to the branches. The tree is drawn to scale, with branch lengths in the same units as those of the evolutionary distances used to infer the phylogenetic tree. The evolutionary distances were computed using the Kimura 2-parameter method [39] and are in the units of the number of base substitutions per site. The analysis involved 56 nucleotide sequences. All positions containing gaps and missing data were eliminated. There were a total of 1241 positions in the final dataset. Evolutionary analyses were conducted in MEGA7 [40]

*S. socius* SMB35 ^T^ (DSM 19940 ^T^) is the type strain of this species with a well-defined status within the family *Halomonadaceae* [9, 10], but the taxonomic placement of strains MH3R3–1 and SMB17 was not characterized in details. We compared partial (1333 bp) 16S rDNA sequences of MH3R3–1 and SMB17 to those of type strains (*n* = 53) representing main phylogenetic groups recognized within the family *Halomonadaceae* [10, 11]. Results of the analysis revealed that the strain SMB17 belonged to the genus *Chromohalobacter* and was closely related to *C. japonicus*, whereas MH3R3–1 was most similar to *Salinicola salarius* and *S. socius* (Fig. 2).

## Genome sequencing information

### Genome project history

Strains *S. socius* SMB35 ^T^, *Salinicola* sp. MH3R3–1 and *Chromohalobacter* sp. SMB17 were selected for genome sequencing on the basis of their phylogenetic position and ability to produce compatible solutes ectoine and hydroxyectoine. Genome sequences provide insights into genetic diversity of halophilic bacteria associated with soils affected by potash mining activity in the Perm region of Russia. Genome sequencing was performed by the AgriLife Genomics and Bioinformatics Service at Texas A&M University (College Station, TX). Draft genome sequences of strains SMB35 ^T^, MH3R3–1, and SMB17 are listed in the Genomes Online Database [12] of the JGI under project IDs Gp0136514, Gp0191572, and Gp0191573, respectively. The corresponding WGS projects have been deposited at GenBank under accession numbers MSDO00000000, MSDP00000000, and MSDQ00000000. A summary of the genome sequencing projects and their compliance with MIGS version 2.0 standards [13] is provided in Table 4.

**Table 4:** Project information

MIGS ID	Property	*S. socius* SMB35^T^	*Salinicola* sp. MH3R3–1	*Chromohalobacter* sp. SMB17
MIGS 31	Finishing quality	Draft	Draft	Draft
MIGS-28	Libraries used	Illumina Paired-End library (300 bp insert size)	Illumina Paired-End library (300 bp insert size)	Illumina Paired-End library (300 bp insert size)
MIGS 29	Sequencing platforms	Illumina HiSeq	Illumina HiSeq	Illumina HiSeq
MIGS 31.2	Fold coverage	302×	326×	318×
MIGS 30	Assemblers	ITMO Genome Assembler v0.1.3	ITMO Genome Assembler v0.1.3	ITMO Genome Assembler v0.1.3
MIGS 32	Gene calling method	RAST	RAST	RAST
	Locus Tag	BTW07	BTW08	BTW10
	Genbank ID	MSDO00000000	MSDP00000000	MSDQ00000000
	GenBank Date of Release	01/05/2017	01/05/2017	01/05/2017
	GOLD ID	Gp0136514	Gp0191572	Gp0191573
	BIOPROJECT	PRJNA357614	PRJNA357619	PRJNA357624
MIGS 13	Source Material Identifier			
	Project relevance	Soil remediation	Soil remediation	Soil remediation

### Growth conditions and genomic DNA preparation

Strains SMB35 ^T^, SMB17, and MH3R3–1 were cultured with shaking at 28 °C in liquid RMM medium. The cells were pelleted by centrifugation and the genomic DNA was extracted using the UltraClean Microbial DNA Isolation Kit (MoBio Laboratories, Carlsbad, CA, USA) according to the manufacturer’s recommendations. DNA concentrations were measured fluorometrically by using a DNA Quantitation Kit (Bio-Rad, Hercules, CA, USA). For each strain, ten micrograms of DNA were diluted to concentration of 200 ng/μl and shipped to the AgriLife Genomics and Bioinformatics Service facility for library construction and Illumina sequencing.

## Genome sequencing and assembly

Samples of purified DNA from strains SMB35 ^T^, MH3R3–1, and SMB17 were used to construct short-insert paired-end libraries with an average insert size of 320 bp, which were sequenced using a HiSeq 2500 instrument (Illumina, San Diego, CA, USA). Raw reads were filtered using a FastQC toolkit [14] followed by assembly with the ITMO Genome Assembler v. 0.1.3 [15]. For SMB35 ^T^, the sequence run produced 2,530,015 × 2 reads totaling 1,265,007,500 bp of data. A *k*-mer length of 53 nucleotides was chosen to optimize for the highest N_50_. The resulting assembly had 164 contigs with a total length of 4.19 Mb (N_50_, 199,518 bp; N_max_, 370,185 bp; median coverage, 302×). Sequencing of the MH3R3–1 sample yielded 2,690,448 × 2 reads that totaled 1,345,224,000 bp of data. The maximum *k*-mer value of 63 was chosen for further analysis since it yielded the best combination of N_50_ and genome length. The final assembly had 159 contigs with a total length of 4,121,883 bp (N_50_, 139,268 bp; N_max_, 370,744 bp; median coverage, 326×). The final assemblies of SMB35 ^T^ and SMB17 genomes were aligned and ordered using progressive Mauve v.2.3.1 [16]. For SMB17, a total of 2,690,448 × 2 reads were obtained that yielded 1,201,941,000 bp of data. The final 3,775,557-bp assembly was based on the *k*-mer value of 63 and consisted of 118 contigs (N_50_, 109,330 bp; N_max_, 468,741 bp; median coverage, 318×) that were aligned and ordered against the finished genome of *Chromohalobacter salexigens* DSM 3043 ^T^ [17].

### Genome annotation

The genomes were annotated using the RAST v.2.0 pipeline [18]. Additional gene prediction analysis and functional annotation were performed using Geneious v.8.1.8 (Biomatters, Auckland, New Zealand) and tools implemented in PATRIC [19] and JGI Integrated Microbial Genomes [20] databases. Putative membrane and secreted proteins were identified using TMHMM v.2.0 [21] and SignalP 4.1 [22] servers, respectively. For SignalP analysis, the Dmaxcutoff value of 0.57 was used for sequences with transmembrane helices and 0.51 for sequences without. Secondary metabolite production clusters were identified using the antiSMASH program [23]. The analysis of the transporter potential was conducted using the Transporter Automatic Annotation Pipeline [24]. Genomic islands were identified and visualized using the IslandViewer 3 [25]. Circular comparative maps of the genomes of the strains SMB35 ^T^, SMB17, and MH3R3–1 were generated using the BRIG software [26].

## Genome properties

The genome of *S. socius* SMB35 ^T^ is comprised of a 4,185,599-bp long chromosome with a 62.2% G + C content. The coding regions accounted for 85.3% of the genome and contained 3,739 protein-coding genes and 89 RNA genes. A total of 2832 genes were assigned a putative function with the remaining annotated as conserved hypothetical or hypothetical. A total of 2037 genes (54.5%) were assigned to COGs. The draft genome of *Salinicola* sp. MH3R3–1 is 4,121,883-bp long with a 62.0% G + C content. The genome has 3620 protein-coding genes and 130 RNA genes. The coding regions accounted for 86.0% of the whole genome and 2903 genes were assigned a putative function with the remaining annotated as hypothetical proteins. A total of 1870 genes (49.9%) were assigned to COGs. The draft genome of *Chromohalobacter* sp. SMB17 is 3,775,557-bp long with a 60.5% G + C content. The genome has 3486 protein-coding genes and 123 RNA genes. The coding regions accounted for 87.0% of the whole genome and 2875 genes were assigned a putative function with the remaining annotated as hypothetical proteins. A total of 1947 genes (54.0%) were assigned to COGs. The properties and the statistics of the three sequenced genomes are presented in Table 5. The distribution of genes into COG functional categories is summarized in Table 6.

**Table 5:** Genome statistics

Attribute	*S. socius* SMB35^T^		*Salinicola* sp. MH3R3–1		*Chromohalobacter* sp. SMB17	
	Value	% of Total	Value	% of Total	Value	% of Total
Genome size (bp)	4,185,599	100.0	4,121,883	100.0	3,775,577	100.0
DNA coding (bp)	3,568,310	85.3	3,547,145	86.0	3,547,145	87.0
DNA G + C (bp)	2,244,356	62.2	2,554,139	62.0	2,284,727	60.5
DNA scaffolds	173	-	159	-	118	-
Total genes	3828	100.0	3750	100.0	3609	100.0
Protein coding genes	3739	97.7	3620	96.5	3486	96.6
RNA genes	89	2.3	130	3.5	123	3.4
Pseudo genes	-	-	-	-	-	-
Genes in internal clusters	-	-	-	-	-	-
Genes with function prediction	2832	74.0	2903	77.4	2875	79.7
Genes assigned to COGs	2037	54.5	1870	49.9	1947	54.0
Genes with Pfam domains	2652	69.3	2609	69.6	2731	75.7
Genes with signal peptides	239	6.4	238	6.6	224	6.4
Genes with transmembrane helices	859	23.0	867	24.0	815	23.4
CRISPR repeats	0	-	0	-	0	-

**Table 6:** Number of genes associated with general COG functional categories

Code	*S. socius* SMB35^T^		*Salinicola* sp. MH3R3–1		*Chromohalobacter* sp. SMB17		Description
	Value	%	Value	%	Value	%	
J	188	5.03	182	5.03	175	5.02	Translation, ribosomal structure and biogenesis
A	159	4.25	157	4.34	164	4.70	RNA processing and modification
K	27	0.72	27	0.75	25	0.72	Transcription
L	111	2.97	107	2.96	106	3.04	Replication, recombination and repair
B	-	-	-	-	-	-	Chromatin structure and dynamics
D	31	0.83	30	0.83	30	0.86	Cell cycle control, cell division, chromosome partitioning
V	62	1.66	73	2.02	67	1.92	Defense mechanisms
T	101	2.70	98	2.71	90	2.58	Signal transduction mechanisms
M	164	4.39	170	4.70	157	4.50	Cell wall/membrane biogenesis
N	73	1.95	72	1.99	64	1.84	Cell motility
U	210	5.62	216	5.97	154	4.42	Intracellular trafficking and secretion
O	82	2.19	88	2.43	103	2.95	Posttranslational modification, protein turnover, chaperones
C	100	2.67	129	3.56	106	3.04	Energy production and conversion
G	365	9.76	375	10.36	421	12.08	Carbohydrate transport and metabolism
E	433	11.58	442	12.21	360	10.33	Amino acid transport and metabolism
F	102	2.73	97	2.68	106	3.04	Nucleotide transport and metabolism
H	234	6.26	188	5.19	253	7.26	Coenzyme transport and metabolism
I	146	3.90	153	4.23	156	4.48	Lipid transport and metabolism
P	129	3.45	104	2.87	134	3.84	Inorganic ion transport and metabolism
Q	6	0.16	6	0.17	4	0.11	Secondary metabolites biosynthesis, transport and catabolism
R	291	4.52	242	6.69	262	7.52	General function prediction only
S	122	3.26	115	3.18	97	2.78	Function unknown
-	1702	45.52	1635	45.17	1539	44.15	Not in COGs

The total is based on the total number of protein coding genes in the genome

## Insights from the genome sequence

**Fig. 3:**
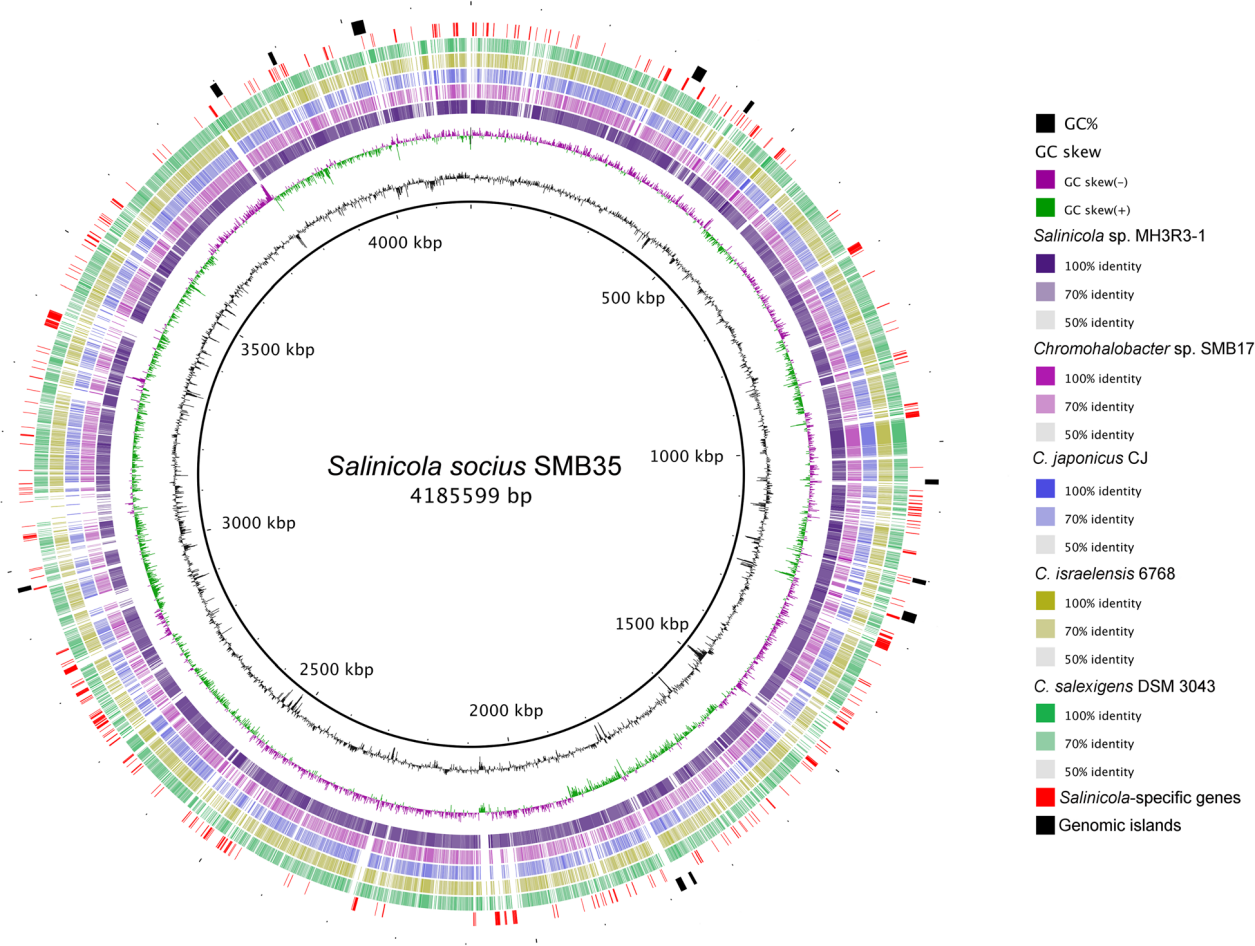
Circular representation of the *S. socius* SMB35^T^ genome and its comparison to genomes of the *Salinicola-Chromohalobacter* group. Rings from inside to outside: (1) GC content, (2) GC skew, (3) BLASTn comparison with *Salinicola* sp. MH3R3–1, (4) BLASTn comparison with *Chromohalobacter* sp. SMB17, (5) BLASTn comparison with *C. japonicus* CJ, (6) BLASTn comparison with *C. israelensis* 6768, (7) BLASTn comparison with *C. salexigens* DSM 3043^T^, (8) Genome segments present in both *Salinicola* genomes, but absent from genomes of all *Chromohalobacter* spp., (9) Genomic islands predicted in the SMB35^T^ genome. The genome comparisons were visualized using the BRIG software [26]. The inner scales designate the coordinates in kilobase pairs

**Fig. 4:**
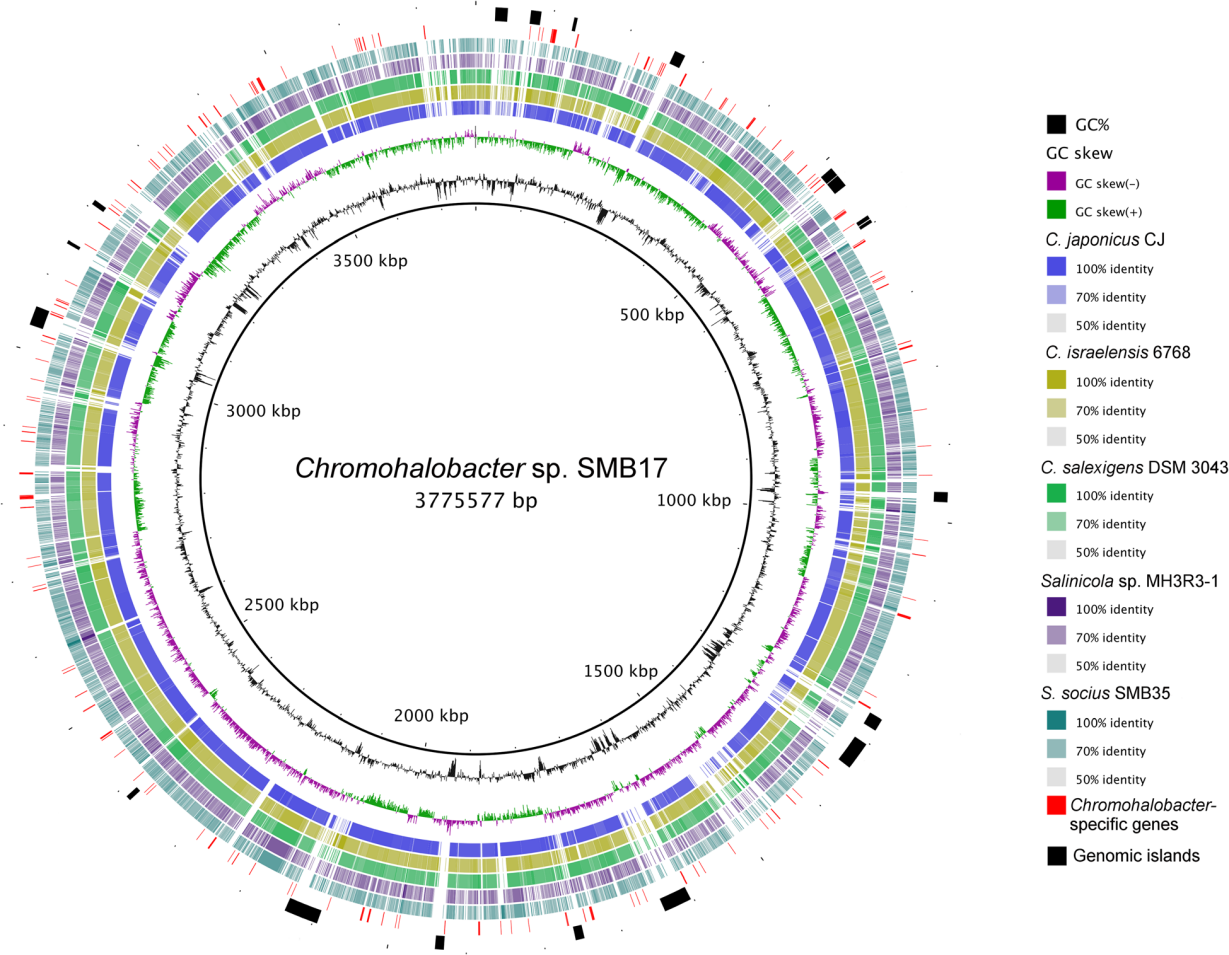
Circular representation of the *Chromohalobacter* sp. SMB17 genome and its comparison to genomes of the *Salinicola-Chromohalobacter* group. Rings from inside to outside: (1) GC content, (2) GC skew, (3) BLASTn comparison with *C. japonicus* CJ, (4) BLASTn comparison with *C. israelensis* 6768, (5) BLASTn comparison with C. salexigens DSM 3043^T^, (6) BLASTn comparison with *Salinicola* sp. MH3R3–1, (7) BLASTn comparison with *S. socius* SMB35^T^, (8) Genome segments present in all *Chromohalobacter* genomes, but absent from genomes of *Salinicola* spp., (9) Genomic islands predicted in the *Chromohalobacter* sp. SMB17 genome. The genome comparisons were visualized using the BRIG software [26]. The inner scales designate the coordinates in kilobase pairs

Phylogenetic analysis revealed that species of *Salinicola* and *Chromohalobacter* constitute two closely related yet distinct clades within the *Halomonadaceae* family (Fig. 2). We compared the newly sequenced *Salinicola* and *Chromohalobacter* genomes to each other and the available genome sequences of closely related strains *C. japonicus* CJ (NZ_CDGZ00000000), *C. israelensis* 6768 (NZ_JQNW00000000), and *C. salexigens* DSM 3043 ^T^ (CP000285). Results of these comparisons revealed that *Salinicola* genomes are larger than those of *Chromohalobacter* by an average of 0.47 Mbp, which amounts to approximately 400 additional protein-coding genes. Our analyses also showed that bacteria of *Salinicola*–*Chromohalobacter* group share a core genome containing 2817 protein-coding genes, which represents 75% to 89.8% of the predicted proteome of each strain (Figs. 3 and 4). Genes conserved among all of the genomes encode proteins contributing to many basic housekeeping functions and physiological mechanisms that allow these organisms to cope with deleterious effects of water stress and thrive in hypersaline environments. The mechanisms of osmotolerance in bacteria involve alterations in the permeability or stability of cytoplasmic membrane, regulation of the concentration of intracellular solutes to adjust the cytoplasm osmolality, and production and uptake of inert metabolites collectively known as compatible solutes, osmolytes, or osmoprotectants. Such compounds help to balance the osmotic pressure across the cellular membrane without compromising protein folding or other cellular processes and include certain polyols, sugars, amino acids, amino acid derivatives, and peptides. The analysis of sequenced genomes revealed multiple pathways predicted to function in the de novo synthesis or uptake of osmoprotectants. Most of these determinants have homologs in the well-characterized strain *C. salexigens* DSM 3043 ^T^ [4] and collectively suggest that strains SMB35 ^T^, SMB17, and MH3R3–1 respond to osmotic stress by accumulating the osmoprotectants ectoines, glycine betaine, and trehalose.

Each of the sequenced strains was predicted to synthesize ectoine from L-aspartate-β-semialdehyde and carried a three-gene operon encoding L-2,4-diaminobutyric acid acetyltransferase 2,4-diaminobutyric acid transaminase and ectoine synthase. Each microorganism also had a gene for the enzyme ectoine hydroxylase, which converts ectoine to 5-hydroxyectoine. However, in contrast to the highly conserved location of the *ectABC* operon, the *ectD* gene was present in different parts of the SMB35 ^T^, SMB17, and MH3R3–1 genomes and not associated with *ectR*, which encodes the transcriptional repressor of the ectoine pathway in *C. salexigens* DSM 3043 ^T^ [2]. In addition to producing ectoines de novo, many bacteria will readily scavenge these metabolites from the environment, and our analyses revealed that all strains featured in this study are capable of taking up ectoines through orthologs of the ectoine-hydoxyectoine ABC-type transport system of *Ensifer meliloti* [27]. The system consists of an ATPase EhuA, a periplasmic solute-binding protein EhuB, and permease proteins EhuC and EhuD that are encoded in genomes of SMB35 ^T^, SMB17, and MH3R3–1 by the highly conserved *ehuABCD* operon. It also appears that the studied strains can utilize ectoines as sources of carbon and nitrogen. The three sequenced genomes share a cluster of ectoine catabolism genes *doeABXCD* that control the degradation of ectoine via hydrolysis to *N*α-acetyl-L-2,4-diaminobutyric acid, deacetylation to diaminobutyric acid and ultimate conversion to aspartate.

In addition to ectoines, members of the *Halomonadaceae* utilize as osmoprotectants glycine betaine and other quaternary ammonium compounds, which are hypothesized to be ubiquitous and relatively abundant in the environment [28]. The three strains share six genes encoding putative BCCT-type transporters, which translocate quaternary ammonium compounds across the membrane using proton/sodium-motive force [29]. The strains also share two gene clusters encoding putative osmoprotectant-specific ATP-binding cassette transporters. The first of these clusters encodes an ortholog of ProVWX, which functions in *Escherichia coli* as a high-affinity uptake system for GB [30]. At lower affinities, the ProVWX complex also transports choline and many other osmoprotectants, including carnitine, proline, taurine, and ectoine. The second gene cluster encodes an ortholog of the OsmF-YehYXW system, which functions in *E. coli* as a low-affinity non-osmoregulatory GB-specific transporter [31]. Finally, the sequenced genomes also share at least three genes encoding proteins similar to GB-specific substrate-binding components of ABC transporters. Like most heterotrophic bacteria, SMB35 ^T^, SMB17, and MH3R3–1 lack genes for the de novo synthesis of GB, but are capable of converting choline to GB using choline oxidase BetA and betaine aldehyde dehydrogenase that are encoded by the *betIBA* operon. The operon also encodes BetI, a choline-sensitive transcriptional regulator of the TetR family of transcriptional repressors. Depending on the environmental conditions, GB can be accumulated as an osmoprotectant or converted to glycine and ultimately catabolized as a source of carbon and nitrogen. The catabolism of GB is a multistep process that starts with the demethylation of GB to dimethylglycine by the GbcAB oxygenase. The resultant dimethylglycine is then converted by a heterodimeric flavin-linked oxidoreductase DgcAB to sarcosine, which is further demethylated to glycine by a heterotetrameric sarcosine oxidase SoxABDG. Genes for all enzymes mentioned above were present in the genomes of SMB35 ^T^, SMB17, and MH3R3–1, along with the AraC-family transcriptional activator GbdR. In other species of bacteria, GbdR senses levels of GB and dimethylglycine and induces transcription of genes involved in the uptake and catabolism of GB, and detoxification of the catabolic byproducts [32].

We further analyzed the sequenced genomes for the presence of genes involved in the synthesis of trehalose, a compatible solute synthesized by many microorganisms in response to stress imposed by high osmolarity, desiccation, heat, freezing, or oxidation [33]. One of the most common ways of synthesizing trehalose by bacteria involves the TPS/TPP pathway, in which the enzyme trehalose-6-phosphate synthase catalyzes the transfer of glucose from UDP-glucose to glucose-6-phosphate, leading to trehalose-6-phosphate [34]. The latter is then dephosphorylated by TPP to release free trehalose. Our analyses revealed that *otsAB* genes encoding enzymes of the TPS/TPP pathway were present in genomes of strains SMB35 ^T^, SMB17, and MH3R3–1, and other bacteria of the *Salinicola*–*Chromohalobacter* lineage. The contribution of the *otsAB* genes to the stress response was demonstrated in *C. salexigens* DSM 3043^T^ where trehalose produced via the TPS/TPP pathway was characterized as a secondary solute involved in osmo- and thermoprotection [35].

We also conducted proteome comparisons to identify genes that are specifically associated with *Salinicola* or *Chromohalobacter* *.* Results of these analyses revealed 399 genes that were present in *S. socius* SMB35 ^T^ and *Salinicola* sp. MH3R3–1, but absent from the genomes of *Chromohalobacter* spp. (Fig. 3). Almost half of these genes (188 genes, or 47%) encoded conserved hypothetical proteins. Among *Salinicola*-specific genes with predicted functions were genes for the alternative trehalose synthesis pathway that involves the conversion of the terminal unit of a glucose polymer from a α-(1–4) to an α,α-(1–1) configuration, and then the release of the resulting trehalosyl moiety as free trehalose [36]. The key enzymes of this pathway, maltooligosyl trehalose synthase and maltooligosyltrehalose trehalohydrolase, are encoded by *treY* and *treZ* genes, which were identified in *Salinicola* strains SMB35 ^T^ and MH3R3–1 but not in genomes of SMB17 and other *Chromohalobacter* strains. In contrast to the TPS/TPP pathway, the importance of the *treY*–*treZ* pathway for the salinity and temperature tolerance of the *Halomonadaceae* remains to be determined. The rest of *Salinicola*-specific genes encoded putative regulatory proteins (transcriptional regulators, a diguanylate cyclase/phosphodiesterase with PAC/PAS sensor domain, and a RpoN-like sigma factor) and different enzymes, some of which constitute pathways for metabolism of phosphonate and the polyamine spermidine. The *Salinicola*-specific transporter proteins were predicted to function in the transport of sodium, potassium, di- and tricarboxylic acids, and provide resistance to heavy metals such as copper, lead, cadmium, zinc and mercury. Interestingly, SMB35 ^T^ also carried the gene for aminocyclopropane-1-carboxylic acid deaminase, which encodes for an enzyme that converts aminocyclopropane carboxylate into ammonia and α-ketobutyrate. In plants, ACC is the immediate precursor of the hormone ethylene that mediates many developmental processes and response to stress. Stressed plants produce excessive amounts of ethylene, which results in smaller roots and delayed aging, abscission, and senescence [37]. Soil bacteria with ACC deaminase lower plant ethylene levels by catabolizing aminocyclopropane carboxylate, which stimulates root growth and improves plant tolerance to biotic and abiotic stress.

We identified a total of 187 genes that were uniquely present in *Chromohalobacter* spp. and had no counterparts in the sequenced *Salinicola* genomes (Fig. 4). A significant proportion (37%) of these genes belonged to the conserved hypothetical category. The genes with defined functions encoded regulators (transcriptional factors and components of two-component signal transduction circuits), components of type IV fimbriae, a VapC-like toxin/antitoxin system, and various enzymes, including subunits of a formate dehydrogenase complex and components of a cyanophycin biosynthesis pathway. Other *Chromohalobacter*-specific proteins were predicted to function in stress response (detoxification of reactive oxygen and resistance to arsenic) or transport phosphate, di-tripeptides, nitrate, branched chain amino acids, and other solutes.

Finally, the three sequenced genomes were screened for the presence of genomic islands using the IslandViewer 3 pipeline [25]. Results of the analysis revealed that *Chromohalobacter* sp. SMB17 had the highest proportion of DNA assigned to genomic islands (252 kbp, or 6.7%) (Fig. 4), which encoded, among other functions, a betaine-binding subunit of ABC transporter, a tricarboxylate transport system, and several O-antigen biosynthesis enzymes. Other features associated with GIs included multiple transposons, a retron element, and seven integrases, three of which were associated with prophages. *Salinicola socius* SMB35 ^T^ and *Salinicola* sp. MH3R3–1 had, respectively, 233.8 kbp and 129.0 kbp of DNA assigned to genomic islands, which represents 5.6% and 3.1% of the genome of each strain (Fig. 3). These GIs encoded multiple conserved hypothetical proteins, various enzymes, transport and regulatory proteins. In addition, each strain harbored a unique assortment of intact and decaying mobile genetic elements. For example, *S. socius* SMB35 ^T^ harbored three prophages that collectively encompassed 120.6 kbp of the genome and contained genes for structural bacteriophage proteins, DNA metabolism enzymes, transcriptional regulators, and lytic enzymes. All three SMB35 ^T^ prophages contained integrase genes and two of these elements were integrated into tRNA-Gly (CCC) and tRNA-Thr (CGT). The genome of *Salinicola* sp. MH3R3–1 contained two islands (35.7 and 24.9 kbp) that contained genes for plasmid replication, partitioning, and conjugation and exhibited similarity to integrative and conjugative elements, a class of hybrid genomic islands with elements of both temperate phages and conjugative plasmids [38].

## Conclusions

This study provides new insights into the genomic diversity of the family *Halomonadaceae* by contributing three genome sequences of moderately halophilic heterotrophic bacteria of the *Salinicola* - *Chromohalobacter* group. The comparative analysis of genomes of *S. socius* SMB35 ^T^, *Salinicola* sp. MH3R3–1, and *Chromohalobacter* sp. SMB17 underscores the importance of two classes of osmoprotectants, ectoines, and quaternary ammonium compounds, to the capacity of these bacteria to tolerate wide ranges of solute concentrations and adapt to hypersaline environments. The sequenced genomes share highly conserved genes for the synthesis of ectoine (*ectABC*) and hydroxyectoine (*ectD*), and conversion of choline to GB (*betIBA*). The three strains also are capable of achieving osmoprotection by accumulating ectoines and quaternary amines via uptake and share an ectoine-specific ABC-type transporter (EhuABCD), two QA-specific ABC-type transporters (ProVWX and OsmF-YehYXW), and several transporters of the Betaine/Carnitine/Choline-Transport family. The three sequenced strains are further capable of utilizing ectoines and quaternary ammonium compounds as sources of C and N and share a cluster of ectoine catabolism genes (*doeABXCD*), as well as three operons (*gbcAB*, *dgcAB*, and *soxABDG*) that control the degradation of GB to aspartic acid. SMB35 ^T^, MH3R3–1, and SMB17 also carry *otsAB* genes that encode enzymes for the synthesis of trehalose, which in this group of bacteria functions as a secondary compatible solute involved in osmotic and thermal protection. Finally, results of this study revealed that *Chromohalobacter* and *Salinicola* share a core genome of approximately 2800 genes, which represents 75–90% of the predicted proteome of each strain. However, each genus also harbored a set of unique protein-coding genes, which was particularly significant in *Salinicola* and amounted to approximately 0.5 Mbp. The genus-specific genome segments encoded enzymes, transcriptional regulators, transporters, conserved hypothetical proteins, and mobile genetic elements. We hypothesize that this flexible gene pool may contribute to the phenotypic diversity of the *Halomonadaceae* and the ability of these organisms to adapt to changing environmental conditions and colonize new ecological niches.

## Abbreviations

ABC transporters: ATP-binding cassette transporters
ACC: Aminocyclopropane-1-carboxylic acid
BCCT: Betaine/Carnitine/Choline-Transport
BLASTn: Basic local alignment search tool for nucleotide sequences
BRIG: Blast ring image generator
COGs: Clusters of orthologous groups of proteins
DSM: DSMZ-deutsche sammlung von mikroorganismen und zellkulturen
GB: Glycine betaine
GI: Genomic island
ITMO: Saint-Petersburg National Research University of Information Technologies, Mechanics, and Optics
JGI: Joint Genome Institute
MIGS: The minimum information about a genome sequence specification
PAC: Motif C-terminal to PAS motifs
PAC/PAS sensor domain: PAS - domain named after the *Drosophila* protein Period (Per), the human arylhydrocarbon receptor nuclear translocator (Arnt), and *Drosophila* Single-minded (Sim)
PATRIC: Pathosystems Resource Integration Center
Pfam: Protein families
QA: Quaternary amine
RAST: Rapid annotation using subsystem technology
RMM: Raymond’s mineral medium
TPP: Trehalose-6-phosphate phosphatase
TPS: Trehalose-6-phosphate synthase
UDP-glucose: Uridine diphosphate glucose
WGS: Whole genome shotgun
